# The impact of funding models on the integration of registered nurses in primary health care teams: protocol for a multi-phase mixed-methods study in Canada

**DOI:** 10.1186/s12875-022-01900-x

**Published:** 2022-11-19

**Authors:** Maria Mathews, Sarah Spencer, Lindsay Hedden, Julia Lukewich, Marie-Eve Poitras, Emily Gard Marshall, Judith Belle Brown, Shannon Sibbald, Alison A. Norful

**Affiliations:** 1 Department of Family Medicine, Schulich School of Medicine and Dentistry, Western Centre for Public Health and Family Medicine, 1465 Richmond Street, Second Floor, Rm 2140, London, ON Canada N6G 2M1; 2 Faculty of Health Sciences, Simon Fraser University, BC Burnaby, Canada; 3 Faculty of Nursing, Memorial University, St John’s, NL Canada; 4 Faculté de médecine et des sciences de la santé, Université de Sherbrooke, Sherbrooke, QC Canada; 5 Department of Family Medicine, Dalhousie University, Halifax, NS Canada; 6 School of Nursing, Columbia University, New York, NY USA

**Keywords:** Mixed-methods research, Research design, Primary care, Nursing, Family practice nurse, Health systems

## Abstract

**Background:**

Family practice registered nurses co-managing patient care as healthcare professionals in interdisciplinary primary care teams have been shown to improve access, continuity of care, patient satisfaction, and clinical outcomes for patients with chronic diseases while being cost-effective. Currently, however, it is unclear how different funding models support or hinder the integration of family practice nurses into existing primary health care systems and interdisciplinary practices. This has resulted in the underutilisation of family practice nurses in contributing to high-quality patient care.

**Methods:**

This mixed-methods project is comprised of three studies: (1) a funding model analysis; (2) case studies; and (3) an online survey with family practice nurses. The funding model analysis will employ policy scans to identify, describe, and compare the various funding models used in Canada to integrate family practice nurses in primary care. Case studies involving qualitative interviews with clinic teams (family practice nurses, physicians, and administrators) and family practice nurse activity logs will explore the variation of nursing professional practice, training, skill set, and team functioning in British Columbia, Nova Scotia, Ontario, and Quebec. Interview transcripts will be analysed thematically and comparisons will be made across funding models. Activity log responses will be analysed to represent nurses’ time spent on independent, dependent, interdependent, or non-nursing work in each funding model. Finally, a cross-sectional online survey of family practice nurses in Canada will examine the relationships between funding models, nursing professional practice, training, skill set, team functioning, and patient care co-management in primary care. We will employ bivariate tests and multivariable regression to examine these relationships in the survey results.

**Discussion:**

This project aims to address a gap in the literature on funding models for family practice nurses. In particular, findings will support provincial and territorial governments in structuring funding models that optimise the roles of family practice nurses while establishing evidence about the benefits of interdisciplinary team-based care. Overall, the findings may contribute to the integration and optimisation of family practice nursing within primary health care, to the benefit of patients, primary healthcare providers, and health care systems nationally.

**Supplementary Information:**

The online version contains supplementary material available at 10.1186/s12875-022-01900-x.

## Background

Improving access to high quality primary health care is a priority, globally. Despite continued investments, Canada still lags behind other Organization for Economic Co-operation and Development (OECD) countries in access to, quality, and cost of primary care [1]. Registered nurses working in collaboration with family physicians and other health care providers (herein referred to as family practice registered nurses; FP-RN) represent a critical part of feasible, affordable solutions to these issues [2, 3]. FP-RN co-managing patient care as healthcare professionals in interdisciplinary primary care teams have been shown to improve access, continuity of care, patient satisfaction and experiences of care, and clinical outcomes for patients with chronic diseases and to be cost effective [4–12].

There is a lack of universally accepted terminology to describe the professional practice of nurses in primary care [13]. Poitras et al. [14] distinguish between three hierarchically linked terms: role, domain, and activity. Role refers to the “function assumed by the nurse modulated by professional norms, a legislative framework, scope of practice and social system” [15]. A nurse’s role is determined by the ensemble of domains, the “sets of activities of the same nature requiring specific knowledge and expertise” [14]. Family practice nursing domains described in the literature include global assessment, episodic and preventative care, health promotion, chronic disease management, pharmaceutical management, paediatric and women’s health, case management, care coordination, collaboration, and practice organisation [3, 6, 15–19]. Activities (also described as tasks or interventions in the literature) are the “actions undertaken by the nurse to help a patient go from a current state of health to [another]” [15].

Within the nursing profession, family practice nursing is viewed increasingly as a distinct discipline that requires specific skills and training [6, 20–23]. Historically, nurses have had little exposure to family practice in their formal training and most nurses who work in family practices do not have prior experience in primary care before starting work in outpatient and primary care settings [24]. Schools of nursing have only recently started to offer specific programmes in family practice nursing [25] but, for most, family practice nursing is embedded within community care courses. Moreover, a distinct set of competency statements that form the basis of training programmes, standards of care, and professional conduct and that describe the skills, knowledge, and attributes for FP-RN has recently been published [26].

Primary health care reforms across Canada in the 2000s introduced a variety of funding models designed to promote interdisciplinary teams with shared responsibilities for managing the care of patients (i.e., co-management [27]). These funding models include global funding, capitation (based on a roster or on a population in a geographical catchment area), and enhanced fee-for-service [17, 28–32].

Studies have consistently shown that funding models influence nurses’ professional practice, the training and skill sets they need, and team functioning [6, 14]. Funding models also influence the amount of time FP-RN spend performing nursing and non-nursing activities (e.g., administrative tasks, making appointments, cleaning, restocking supplies [19, 33, 34]), the range of activities, and the relative autonomy of nurses and scope of practice. For example, a survey of FP-RN in capitation-funded practices in New Zealand found that nurses spent less than a third of their time performing independent work (i.e., consultations, triage, chronic care management) and roughly two-thirds of their time completing administrative work or activities delegated by a physician [33]. Meanwhile, traditional and enhanced fee-for-service narrow FP-RN activities to billable clinical procedures [21, 34–36] and/or through standing orders or medical directives [19, 20, 34, 35]. In globally-funded clinics, FP-RN appeared to have much greater autonomy; the nurse’s practice was self-determined by professional and regional policies, rather than another health professional [35]. Additionally, FP-RN saw patients and scheduled appointments independently, saw patients in a greater variety of community-based settings, and performed activities targeting broader social determinants of health [35].

Studies have further examined team functioning in the interdisciplinary primary health care teams implemented in Canada in the 2000s, and have identified factors that promote effective team functioning: trust between providers [6, 22, 24, 34, 36–40], shared access to physical resources (space and equipment) [18, 23, 24, 36, 38–41], dedicated time for team meetings [24, 33, 39, 40], access to shared electronic medical records [23, 24, 36, 38–40], role clarity [20, 22, 24, 38–40], medical governance [14, 20, 42, 43], and strong leadership [23, 24, 38–40]. Funding models have been shown to influence these factors. For example, in traditional fee-for-service practices, communication between the physician and nurse was primarily through the medical chart [35]. In enhanced fee-for-service practices in Quebec, communication tended to be from nurse to physician and more consultative than collaborative [20]. In globally funded clinics, physicians and nurses consulted each other as needed, and communicated through case management and hallway conversations [20].

Despite the recognition that funding models are critical to supporting collaborative primary health care teams, there is a lack of evidence explaining how these funding models affect the roles and functions of non-physician health care providers, such as FP-RN [37, 41]. It is further unclear how well these funding models integrate FP-RN into existing (i.e., predominately fee-for-service) primary health care systems. As a result, FP-RN are frequently underutilised and miss opportunities to contribute to high-quality patient care, despite forming the core of interdisciplinary primary health care teams across many Canadian jurisdictions [16, 44, 45].

### Objectives

This research seeks to explore the relationship between funding models and nursing professional practice, the skills and training needed by FP-RN, team functioning, and co-management of patient care in primary health care settings. The goal of this project is to further the integration of FP-RN in primary health care. Specifically, our objectives are to:

1. Describe and compare the various financial models used in Canada to integrate FP-RN in primary health care.
2. Explore the variation of nursing professional practice, training, and skill set needed by FP-RN and team functioning in primary health care settings funded by traditional fee-for service, enhanced fee-for-service, capitation, and global funding in British Columbia, Nova Scotia, Ontario, and Quebec.
3. Examine the relationship between funding model, variation in nursing professional practice, training, and skillsets needed by FP-RN, team functioning, and patient care co-management of FP-RN in primary health care settings in Canada.

We hypothesise that funding models that are linked to the activities of all team providers and have interdependent provider remuneration will promote a broader range of independent activities, require broader training and skill set, promote better team functioning, and produce better co-management compared to funding models that are linked to the activities of a single provider and have hierarchically dependent provider remuneration.

## Methods

### Overall study design

This project employs a multi-phased, mixed-methods design [39] and consists of three linked studies: (1) a funding model analysis, (2) case studies, and (3) an FP-RN survey.

#### Nursing Role Effectiveness Model

**Fig. 1:**
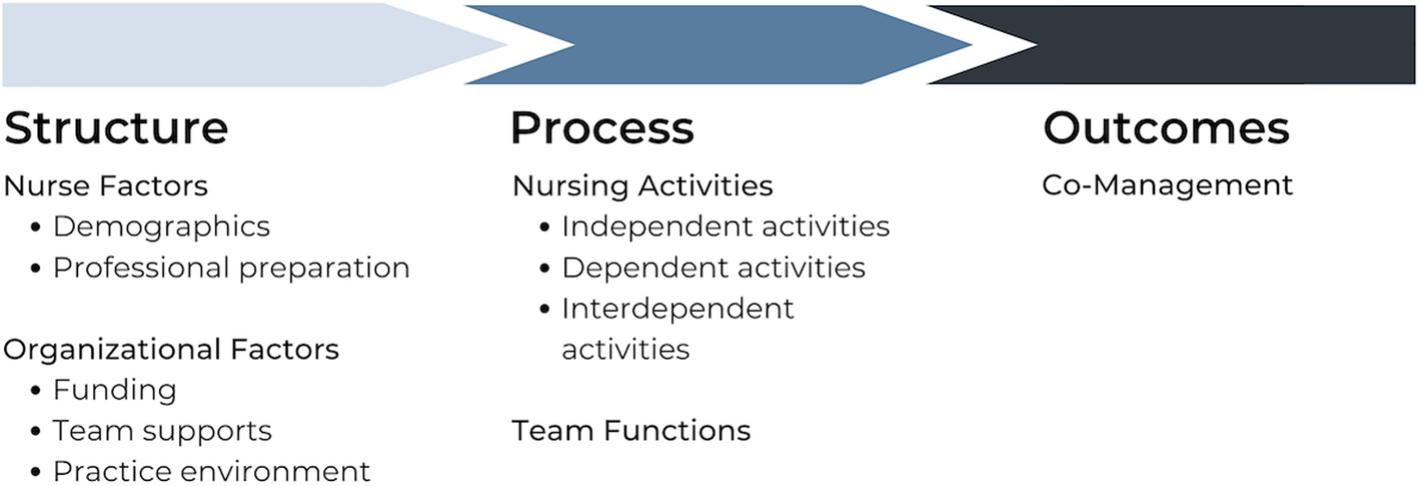
Adapted from the Nursing Role Effectiveness Model [46]

This project uses the Nursing Role Effectiveness Model (Fig. 1) [10, 46], an adaptation of Donabedian’s classic structure-process-outcomes model of quality of care [40], to examine the relationship between the nurse and organisational factors (structure), professional practice of FP-RN and team effectiveness (process), and patient care co-management (outcomes). The Nursing Role Effectiveness Model characterises FP-RN’s professional practice activities as independent, dependent, or interdependent [10]. Nurses alone are accountable for independent activities that are autonomous, nurse-initiated, and carried out without a physician’s order (e.g., triage, patient assessment, and evaluation). Dependent activities are initiated by physician’s orders using the nurse’s clinical judgement (e.g., implementing and coordinating care). Interdependent activities are carried out by nurses along with other health care providers, where each provider has a unique contribution to the activity (e.g., care coordination, quality improvement, team communication).

#### Typology of Financial Models

**Fig. 2:**
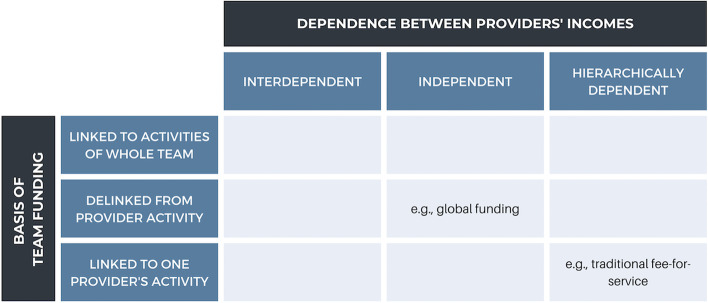
Adapted from the Typology of Financial Models [47]

This project will also employ the Typology of Financial Models [47] to classify and compare funding models. The Typology of Financial Models describes funding models by the degree to which team funding is linked to activities of team members and the interdependence of an individual provider’s remuneration on other providers (Fig. 2). The model captures the interplay between the structure of team funding and team functioning. Team funding is described as “linked”, “delinked”, or “linked to one provider”, usually the family physician. Interdependence of provider remuneration is categorised as “interdependent”, “independent”, or “hierarchically dependent”. Interdependence is present where all providers’ income influences that of their team members; independent remuneration is where providers’ incomes are not dependent on other team members’ incomes; and hierarchical dependence is where one or more provider’s income influences that of other team member, but this dependence is not reciprocal. The typology provides a useful and unifying framework with which to compare different funding models in Canada. In this project, we will use this framework to characterise and categorise funding models.

### PHASE 1: Funding model analysis

To identify relevant funding models, we will review websites of provincial/territorial departments of health, nursing organisations (e.g., unions, regulators), and physician unions (e.g., Ontario Medical Association). We will conduct Google, database, and website searches using combinations of the following terms until search results are exhausted: province or territory name; “primary care” or “family medicine” or “primary care team” or “primary care network” or “team-based care” or “interdisciplinary team”; “family practice nurs*” or “primary care nurs*” or “registered nurse”; “funding model” or “practice model”. We will contact officials in relevant organisations to identify funding and policy documents that are not publicly available and to verify the data collected. We will also search for relevant documents (e.g., published articles, government reports) that examine funding models for FP-RN and interdisciplinary teams (including other nursing professionals who may work in primary care settings such as nurse practitioners, licensed practical nurses, and registered psychiatric nurses). We will use our team members’ networks of primary health care, nursing policymakers, and researchers to identify potential funding programmes and policies.

After an initial scan for results from all provinces and territories in Canada, we will screen the results returned by the string term, snowball, and targeted search results to eliminate duplicates. We will also screen for document relevancy in accordance with inclusion and exclusion criteria. We will include current funding models designed to include FP-RN in primary health care settings, including for specialised primary health care clinics (e.g., chronic disease programme) or clinics targeting specific populations (e.g., Indigenous, student, immigrant, or refugee). Funding models can be either specific to FP-RN or interdisciplinary teams so long as an FP-RN is a health professional eligible to practice within the interdisciplinary team and other inclusion criteria are met.

We will exclude funding models that have been phased out (i.e., no longer fund a single practice in the province) or fund clinics or programs where the primary focus is not primary health care, such as public health, home care, hospital-based ambulatory care, or inpatient care settings. We will exclude funding models for clinics that are specifically designed for nurses with graduate level nursing education beyond that of a typical registered nurse (e.g., nurse practitioner, clinical nurse specialist, northern/remote community health nurse); however, these policies will be included in the analysis of contextual factors described below. Funding models for clinics that require primary health care relevant postgraduate training (e.g., certification in family practice, diabetes educator) will be included.

For each funding model, we will review available documents to describe the following parameters: the province or territory, nature of clinic funding (e.g., fee-for-service, enhanced fee-for-service); the basis for funding level (e.g., roster, geographic area, activity level); type of remuneration for individual providers in a typical clinic (e.g., salary, billings); source of remuneration for individual providers in a typical clinic; interdependence between provider incomes; and supports for team work (e.g., funding for administrator, space, equipment, team based communication, electronic medical record). We will also document eligibility conditions, the estimated number of clinics supported by the funding model, the year the funding model was introduced, and any other contextual factors (e.g., union membership). Funding models will be summarised thematically along parameters identified and categorised using the Typology of Financial Models [47].

### PHASE 2: Case studies

The Case Studies consist of qualitative interviews and FP-RN activity logs.

#### Qualitative interviews

We will interview one or more FP-RN, one physician, and one administrator (if applicable) from each clinic site using semi-structured interview guides tailored to each professional (see Additional file 1). We will ask FP-RN about current nursing professional practice (roles; domains; and independent, dependent, and interdependent activities), nature of teamwork, barriers/facilitators to maximising their scope of practice and teamwork, and any training and education received prior to their position in their current clinic team or since joining the clinic team, and any training or education that has been of particular utility in their current position. We will ask all participants (FP-RN, physician, and administrator) to describe the clinic funding, remuneration of individual providers, linkage of team funding to team members' activities, and interdependence of individual provider remuneration on other providers. The questions on clinic funding will not only be used to describe the funding model and place models in the typology but will also allow us to identify appropriate terminology for the FP-RN surveys. We will also gather relevant demographic data (gender, profession, years of experience, training) to describe participants and clinic-specific data (e.g., number and type of other health providers in clinic, focused versus general practice, specific patient populations, access to space and equipment, dedicated time for team meetings, shared electronic medical records, access to continuing education opportunities) to describe the clinic. Interviews will be conducted by phone or Zoom videoconference based on participant preference, will take roughly one hour, be audio recorded, and will be transcribed for analysis.

#### Nursing activity log

Each FP-RN at each site will be invited to complete an online daily activity log over the course of two weeks (ten working days). We will request FP-RN’s email addresses to send the initial link to the study invitation and daily reminders for ten consecutive working days. To ensure that each FP-RN’s responses are attributed to the appropriate clinic, the initial email will include a study site identification number that each FP-RN will be asked to enter.

The daily activity log will ask each FP-RN about the total number of hours worked each day and the total number of hours spent on independent, dependent, and interdependent activities, as well as non-nursing activities (cleaning or clerical work), and amount of time spent in different locations (clinic, patient home, other community-based setting). Examples of each category of activity, identified through the interviews and the literature, will be provided in the activity log to assist nurses in completing the log.

#### Study sample and recruitment

We will conduct 3–4 case studies of each funding type in each province. Specifically, we will conduct case studies of 6–8 practices funded by traditional fee-for-service (Ontario and Nova Scotia), 9–12 practices funded by enhanced fee-for-service (Ontario [Family Health Groups], Quebec, and Nova Scotia), 3–4 practices funded by capitation (Ontario [Family Health Organisations/Family Health Networks]), 10–12 practices globally funded (British Columbia [Community Health Centres], Ontario, Nova Scotia), and 3–4 practices funded by salary (British Columbia [Primary Care Networks]). We will interview one or more FP-RN, one physician, and one administrator (where applicable) at each site for roughly 96–122 interviews. We anticipate there will be an average of two FP-RN per site for a total of 64 FP-RN completing a Nursing Activity Log as the second component of the case studies.

To identify sites, we will use existing public lists of teams and practices (e.g., Family Health Teams, Community Health Centres, Family Medical Groups, Primary Care Networks, family medicine teaching sites); research datasets available to researchers (e.g., Co-Investigator Marshall’s MAAP dataset in Nova Scotia [48]); the Ontario, Quebec, and Nova Scotia chapters of the Canadian Family Practice Nurses Association; the network of academic family practices at Western, Queen’s, and Dalhousie universities, Université de Sherbrooke, and University of British Columbia; and social media.

To recruit clinic teams, the research coordinator will first reach out to individuals who express their interest in participating (administrator, physician, and/or FP-RN) with an invitation to participate. The invitation will describe the study, ask the individual to share the study information with other potential participants with whom they work in the clinic, and contact the research coordinator if the clinic team is willing to participate in a case study. Recruitment will continue until we have reached saturation.

#### Analysis

Using a thematic analysis approach, at least two members of the research team will independently read each transcript of the qualitative interviews to identify key words/codes and develop a robust coding and analysis template [49–53]. This template will be used to code the transcripts in NVivo V.12 (QSR International). Through various iterations of the coding process, we will move from more descriptive to more analytic codes, developing broader conceptual themes from the interview data. We will compare across funding models to identify key similarities and differences. Transcripts from Quebec will be analysed in French by bilingual members of the team and only quotations appearing in final documents will be translated. Descriptive statistics will be used for the demographic data to summarise the characteristics of study participants.

Using SPSS 27 (IBM SPSS Statistics) to analyse the nursing activity logs, we will tabulate the average amount of time nurses in each funding model spends on different nursing activities. While the sample size is too small to allow complex statistical analyses, the daily activity logs will provide more reliable data than if nurses were asked to estimate amount of time spent on different activities over an imagined “typical” day or week. We will then compare the logs with the activity data from the interviews to assess whether nursing activity variations measured by the activity log triangulate with interview findings.

### PHASE 3: Family practice nurse survey

We will conduct a cross-sectional, online survey of FP-RN in Canada using Qualtrics (SAP Software Solutions). The survey will gather structure, process, and outcome variables from the Nursing Role Effectiveness Model [10]. The survey questionnaire will be informed by the funding model analysis, interviews in the case studies, and consultation with research team members.

Structural factors include FP-RN, practice-specific, and contextual factors. For FP-RN factors, the survey will gather demographics (i.e., age, sex, gender) and professional preparation (i.e., nursing qualification, education, years nursing experience, years of family practice nursing experience, years at current clinic). Practice-specific factors refer to variables specific to the FP-RN’s specific setting (e.g., number and type of other health providers in clinic, focused versus general practice, specific patient populations, access to space and equipment, dedicated time for team meetings, shared electronic medical records). Contextual factors refer to variables that extend beyond the FP-RN’s specific practice setting (e.g., province, funding model, size of community, rurality). To allow us to categorise clinic funding using the Typology of Financial Models, funding model questions will include type of funding, basis for funding level, and dependence between provider incomes.

To capture process factors (i.e., FP-RN activities, team functioning), we will use a modified FP-RN’s activity log developed in the case studies (objective 2) and ask FP-RN to recall their last week at work. FP-RN will be asked about the total number of hours worked during the week and the total number of hours spent on independent, dependent (i.e., delegated), and interdependent patient care activities; non-nursing activities (e.g., cleaning or clerical work); and the amount of time spent in different locations (e.g., clinic, patient home, other community-based setting). Team functioning will be measured using the Team Climate Inventory (TCI) that has been widely used in health care settings, including primary health care, to assess team effectiveness [54–56]. Higher scores on the TCI are correlated with better patient and worker outcomes. The TCI has been validated in English and French and has high internal consistency scores [57].

The outcome (co-management) will be measured using the Provider Co-Management Index (PCMI), a 20-item instrument that is designed to assess the degree to which two primary health care disciplines (e.g., medicine and nursing) share responsibility for all clinical and administrative tasks needed to manage the health care of a given patient [27]. The PCMI consists of three subscales with demonstrated high validity and internal reliability consistency: effective communication (α = 0.811); mutual respect and trust (α = 0.746); and shared philosophy of care (α = 0.779) [58].

#### Study sample and recruitment

Based on published reports, there are roughly 2,000 FP-RN in Canada. Assuming a conservative response rate of 22.5%, we anticipate a sample of 450 completed surveys. Previous surveys of FP-RN have response rates ranging from 18 to 91% [19, 21, 59]. Assuming we will have four funding models with an equal number of respondents (*n* = 125), this sample size will allow us to detect a difference of 0.8 on the TCI and 22% difference in time spent on specific activities (accounting for four groups, an alpha of 0.05, and power of 0.8) [60]. We will assess the representativeness of the sample by comparing respondents’ and population characteristics based on data available on Canadian Family Practice Nurses Association membership statistics.

We will use a modified Dillman approach [61] to conduct the electronic survey. Canadian Family Practice Nurses Association and its affiliated provincial groups and provincial nursing colleges (regulators) will be asked to distribute the survey to their members by e-mail. They will send an initial email inviting members to take part in the study, along with reminders two and four weeks later. Each email will contain a link to the survey. We will offer a lottery incentive (a draw for three $100 gift certificates) to increase response rates. To identify potential duplicate responses, we will include survey items (e.g., day of birth) that, when combined with other survey questions (e.g., year of graduation and province) will generate a quasi-unique identifier.

#### Analysis

Using SPSS, we will use bivariate tests (ANOVA, chi-square) and multivariable regression to examine the relationship between funding model and structure, process, and outcome variables. We will also employ exploratory factor analysis to validate the use of the PCMI among FP-RN in Canada (and the French and English versions of the instrument) [57, 62].

### Sex and gender-based analyses

While nursing activities and the nursing profession are gendered (that is, historically considered women’s roles and professionally dominated by women) [42], there is little data to support gender-based analyses in this project. We will be collecting data on participant gender in the case studies and surveys, but our analyses using these variables will largely be descriptive and hypothesis generating. For example, in the case studies, we will ask whether the gender of physicians and nurses who work closely together influence the activities performed by the nurse (e.g., when working with male physicians, do female nurses perform different activities than when working with female physicians).

### Study rigour

We will take several steps to enhance the rigour of our study [48, 50, 52]. For the case study, we will prepare interview guides and pre-test questions, document interviewing and transcription protocols, train interviewers, and member-check with the participants during interviews. We will keep detailed records of the interviews (transcripts and audiotapes), field notes, drafts of the coding template, and coding disagreements and their resolutions. We will look for negative cases and encourage and document self-reflection among all members of the research team. We will provide thick descriptions and use illustrative quotes.

Prior to distributing the FP-RN survey, we will conduct face and content validity testing with a group of researchers and primary care clinicians to confirm that the survey captures intended concepts and activity data specific to the study sample and variables [63]. We will take steps to validate the PCMI in the French language using forward-backward translation methodology by expert translators, clinicians, and researchers [64]. The survey will then be pre-tested on a group of 8–10 FP-RN and psychometric properties will be calculated to ensure validity and reliability (e.g., Cronbach’s alpha).

Overall, this project will be conducted by an interdisciplinary team of established and early career researchers, FP-RN, family physicians, and provincial policy makers and includes executive members from the Canadian Family Practice Nurses Association. The members of the team have expertise in primary health care, interdisciplinary teams, mixed methods research designs, qualitative interviewing, policy analysis, health economics, case studies, survey design and instrument validation, quantitative and qualitative analysis, and knowledge translation. This allows us to draw on individuals’ expert knowledge in the development of our research tools and interpretation of results.

### Ethical considerations

Written informed consent will be obtained from case study and survey participants prior to their participation in any study procedures. In the case of both interviews and online surveys, individuals may refuse to participate, refuse to answer any questions, or withdraw from the study at any point up until their data have been combined with other participants’ data for analysis. Participants do not waive any legal rights by signing the consent form.

Participants’ full name, email address, and phone number will be used to schedule the interview but this information will be kept separate of their study data and replaced with a unique ID number. Participants’ gender and information about their practice will be used to ensure variation in interview participants. All other identifying information that may be mentioned will be obscured during the transcription process. No identifying information will be shared with team members in other regions. If the results are published, participants’ names will not be used.

### Knowledge translation

As part of our ongoing integrated knowledge translation plan, we will use in-person meetings, videoconferences, and email for ongoing communications between team members. We have strong links with nursing organisations and networks of health workforce and primary health care researchers and knowledge users to inform our research and share findings. Drafts of reports will be distributed to a group of knowledge users from key organisations (e.g., provincial departments of health; the Canadian Family Practice Nurses Association) for comments prior to revising for broader dissemination.

Our end-of-grant knowledge translation goals are to disseminate findings to inform policy and programme discussions, encourage further research, and raise public awareness of study findings. We will conduct a series of stakeholder sessions to engage policy makers with the data and provide opportunities for them to think through what the findings might mean for them. To facilitate participation, in-person sessions will be held in conjunction with existing events (e.g., Canadian Health Workforce Conference, Canadian Family Practice Nurses Association biennial conference or educational webinar series). Summary reports for each study and the synthesis will be written for decision-makers. These reports will be shared with these target audiences and made available through the Canadian Family Practice Nurses Association and the Canadian Health Workforce Network websites. To reach academic researchers and knowledge users interested in primary health care and nursing workforce issues, we will present at regional, national, and international conferences. We will prepare articles for publication in peer-reviewed open access journals, and technical reports detailing methods and sensitivity/supplementary analyses. To reach the public, we will write op-eds, conduct media interviews, participate in on-line discussions (e.g., healthydebate.ca), and utilise social media.

## Discussion

This project represents a critical step toward integrating and optimising family practice nursing within primary health care and will benefit patients, primary healthcare providers, and healthcare systems nationally. This project will address a gap in Canadian literature and aid provincial governments in structuring funding models that best optimise the roles of FP-RN and realise benefits from team-based care [41].

The results of the project will shed light on how funding models impact healthcare services and optimal professional practice deployment. The results will be useful to provinces that have already introduced funding reforms to integrate FP-RN into primary health care settings as well as provinces (such as Newfoundland and Labrador) that have yet to introduce similar reforms. The project will inform the training of the family practice nursing workforce by linking the professional practices, training, and skill sets of FP-RN to different funding models. These findings will be especially useful as nursing schools in Canada integrate family practice nursing into their curricula and/or expand family practice certification programmes.

By incorporating the Nursing Role Effectiveness Model [10], the results of the study contribute to evaluating the integration of family practice nurses in primary health care [10, 28, 45, 59, 65–68]. Funding models have important mediating effects that shape both what nurses do, and the health and health system outcomes they produce [10]. By developing new ways of measuring nursing professional practice and co-management, the project contributes to the development of robust metrics to evaluate the outcomes attributable to FP-RN.

### Limitations and challenges

Though this study involves a national survey of FP-RN, the case studies are limited to four provinces. These four provinces have adopted different funding models and approaches to integrating team-based care, providing opportunities for comparison across regions and models. Due to the heterogeneity of health systems, reforms, and variation in how the same funding model is applied in other Canadian provinces and health systems outside of Canada, case study findings may not be generalisable beyond the study provinces. Additionally, depending on the power of the FP-RN survey results, we may not be able to fully extricate the interaction between provinces and funding models.

FP-RN who participate in case studies will complete activity logs at the end of each workday, which may be susceptible to recall bias. The data from these logs, however, will be cross-referenced and interpreted in conjunction with qualitative interviews and FP-RN survey results. This study is also focused on health care providers perspectives and will not account for patients’ experiences with family practice nurses that could be informative for understanding FP-RN’s roles and integration in clinical care, the sufficiency of current FP-RN practice competencies, and any needs for future training.

## Supplementary Information

**Additional file 1.** Case Study Interview Guides.

## Data Availability

Not applicable.

## Abbreviations

FP-RN: family practice registered nurse
PCMI: Provider Co-Management Index
TCI: Team Climate Inventory
