# Supplementary material for: The impact of funding models on the integration of registered nurses in primary health care teams: protocol for a multi-phase mixed-methods study in Canada
Source: BMC Prim Care. 2022 Nov 19;23:290. doi: 10.1186/s12875-022-01900-x (PMC9675973; doi:10.1186/s12875-022-01900-x)
Supplement: Supplementary file 1 — Additional file 1. Case Study Interview Guides. [file 12875_2022_1900_MOESM1_ESM.docx]

**ADDITIONAL FILE 1**. CASE STUDY INTERVIEW GUIDES

Family Practice Registered Nurses

*Thanks for taking the time to speak with me today. First, I’d like to tell you a little bit about the study. As you may know, the role of registered nurses within family practice settings has not been well established, therefore, the goal of this interview is to learn more about registered nurses in family practice settings across the province. We will be asking you questions about funding, nursing professional practice such as your roles, domains and activities, the skills and training needed in your practice, primary care team functioning, and the co-management of patient care. It should take us about an hour. I’d like to record our interview today, just so that I don’t miss anything you say. Is that ok with you? I want to let you know that your identity will be kept confidential, and your name will never be used in any kind of reporting. You may end the interview at any time or choose not to answer any specific question. Did you have any questions about anything before we get started?*

First, I would like to collect some basic demographic information.

1. How long have you been practicing as a family practice Registered Nurse?
2. How long have you been employed at this primary care setting?
3. What was your previous nursing experience?
4. In terms of gender, do you identify as male female, non-binary or something else?

In the next set of questions, I’d like to focus on your current nursing professional practice within the clinic. We will be discussing your nursing and non-nursing activities, your range of activities and the nature of teamwork within your clinic.

1. Can you tell me about what a typical day for you is like in this clinic?

- *[Probe based on responses to first set of questions]* global assessment, episodic and preventative care, health promotion, chronic disease management, pharmaceutical management, paediatric and women’s health, case management, care coordination, collaboration, and practice organisation.

1. Does your clinic offer specialised primary health care programs (e.g., chronic disease program) or target specific populations (e.g., Indigenous, student, immigrant, or refugee)?
2. Who are other members of the primary care team? (e.g., number of other providers and roles)
3. How is your current position within the primary care setting funded?
   Does the physician pay your salary directly?
   Does your salary dependent on the physician’s salary or vice versa?
   Are you employed at the clinic full-time, part-time, or casually?

- *[Probe based on responses to first set of questions]* (Enhanced) Fee for Service, Capitation, globally funded

1. Could you describe your independent activities, if any? This refers any activities which are autonomous, nurse-initiated, and carried out without a physician’s order such as triage, patient assessment, and evaluation.

- *[Probe based on responses to first set of questions]*

1. Could you describe your dependent activities, if any? These are activities initiated by physician’s orders using your clinical judgement such as implementing and coordinating care. For e.g., wound care

- *[Probe based on responses to first set of questions]*

1. Could you describe your interdependent activities, if any? These are carried out by RNs along with other health care providers, where each provider has a unique contribution to the activity. For e.g., care coordination, quality improvement, team communication.

- *[Probe based on responses to first set of questions]*

1. Considering independent, dependent, and interdependent activities, which of these activities encompass most of your workday?
2. How much of your time in the clinic is dedicated to nursing activities compared to non-nursing activities (e.g., administrative tasks, making appointments, cleaning, restocking)?
3. How did you and the physician/team arrive at an agreement about your role in the clinic?
4. How do you feel about your scope of practice in this role? How do you feel about the use of your knowledge/skills in this role?
5. What resources are available to you to help you work within this clinic?

- *[Probe based on responses to first set of questions]* meeting space, access to EMR, private office space

1. How has your education prepared you to work within this clinic?
   Have you received any training and education prior to your current position?
   What training and education have you received since beginning your position at the family practice clinic?
   Were these particularly useful in your current position?

- *[Probe based on responses to first set of questions]* certification in family practice, diabetes educator

1. What gaps do you see in your education for working in this setting?
   Are there gaps in knowledge/skills specific to this role within the clinic?
2. Do you feel that you are maximizing your potential contributions to patient care in this clinic?
   What do you find prevents/hinders you from optimizing your contribution to patient care within this clinic?
   If your role is already optimized, what are some facilitators?

- *[Probe based on responses to first set of questions]* *Barriers:* lack of time, cost of continuing education, inability to order diagnostic tests, lack of space, equipment or supplies, inability to prescribe/revise medications, lack of support from other providers, underutilized clinical skills, physicians attitudes towards role, patients perception of role, lack of educational opportunities, lack of clear job description; *Facilitators*: collaboration with physicians/other healthcare providers; access to education/training, positive patient feedback, supportive employment conditions, availability of space, equipment or supplies, ability to practice autonomously, awareness and access to programmes for clients, collaboration with community services

1. How does your health care team coordinate patient care?
   What supports are available to you to help carry out your roles? What are some barriers?

*[Probe based on responses to first set of questions]* improve access, continuity of care, patient satisfaction, clinical outcomes for patients with chronic diseases

1. Could you describe any efforts put into facilitating teamwork within your health care team? What are some barriers to optimizing teamwork?

*[Probe based on responses to first set of questions]* dedicated time for team meetings, team emails

For the final question, I’d like to switch gears a bit.

1. Thinking of your gender…Have you ever felt your gender influences the roles that you are able to play in the clinic?
   Are your nursing activities influenced by the gender of the physician?

- Probe: Admin work, independent work, dependent work

1. Those are all the questions I have. Is there anything you would like to add?

Family Physician

*Thanks for taking the time to speak with me today. First, I’d like to tell you a little bit about the study. As you may know, the role of registered nurses within family practice settings has not been well established, therefore, the goal of this interview is to learn more about registered nurses in family practice settings across the province. We will be asking you questions about clinic funding, primary care team functioning, and the co-management of patient care. It should take us about an hour. I’d like to record our interview today, just so that I don’t miss anything you say. Is that ok with you? I want to let you know that your identity will be kept confidential, and your name will never be used in any kind of reporting. You may end the interview at any time or choose not to answer any specific question. Did you have any questions about anything before we get started?*

First, I would like to collect some basic demographic information.

1. How long have you been practicing as a family physician?
2. How long have you been employed at this primary care setting?
3. What was your previous experience as a physician?

In the next set of questions, I’d like to focus on the roles of your nurses within the clinic. We will be discussing how their nursing activities are determined, collaboration between yourself and your nurses on patient care, and the nature of teamwork within your clinic.

1. Can you tell me about the role of the family practice nurse within your setting?
   To what extent does the nurse practice autonomously?
   To what degree is the nurse involved in care planning and decisions?
   What types of populations does the nurse serve?
2. Do you feel that the nurse is maximizing his/her/their potential contributions to patient care?
3. Was there anything done to prepare the physician(s), clinic staff and patients for the registered nurse’s arrival? If yes, please specify.
   Was there anything that could have been done to help facilitate the integration of the registered nurse into the practice?
   What would have helped the transition for the physician(s), clinic staff, patients, and the new employee (i.e., registered nurse)?

OR

Can you tell me about any strategies, whether formal or informal, that were implemented to facilitate collaboration between nurses and physicians/staff at your clinic?

1. Can you tell me about the nature of the collaborative practice within your primary care setting? How does the family practice nurse relate to/interact with other healthcare providers within and outside of your setting (including yourself)? What are the nurse’s roles in collaborative practice (e.g., referrals, chronic disease management)
2. Are there other healthcare providers within the setting? (number of providers and role)
3. What gaps do you see in basic education for nurses working in this setting? Are there any particular areas where you have identified learning needs? Tell me about how it affects your practice.
4. What additional training would support the nurse to work optimally within your practice?
5. How is the family practice nursing position funded?
6. What do you perceive as barriers to optimizing the family practice nurse’s contributions to patient care within this primary care setting? What do you perceive as facilitators to optimizing the family practice nurse’s contributions to patient care within this primary care setting?
7. Do you have any additional information that you would like to share?
8. Lastly, you may have seen on the consent form that there is a $100 honorarium for interview participants which we can send to you as an e-gift card to a business of your choosing that we would send directly to your email.
   1. Is there a preferred business for which we can provide you a gift card?
   2. Would you like the gift card sent to the email which we used to schedule this interview or do you have an alternative email that you would prefer?

Clinic Administrator

*Thanks for taking the time to speak with me today. First, I’d like to tell you a little bit about the study. As you may know, the role of registered nurses within family practice settings has not been well established, therefore, the goal of this interview is to learn more about healthcare team function including registered nurses and administrators in family practice settings across the province. We will be asking you questions about clinic funding, primary care team functioning, and the co-management of patient care. It should take us about an hour. I’d like to record our interview today, just so that I don’t miss anything you say. Is that ok with you? I want to let you know that your identity will be kept confidential, and your name will never be used in any kind of reporting. You may end the interview at any time or choose not to answer any specific question. Did you have any questions about anything before we get started?*

First, I would like to collect some basic demographic information.

1. How long have you been practicing as an administrator?
2. How long have you been employed at this primary care setting?

In the next set of questions, I’d like to focus on your roles within the clinic. We will be discussing how your activities within the clinic are determined, collaboration between yourself and your healthcare team on patient care and the nature of teamwork within your clinic.

1. Can you tell me about what a typical day for you is like in this clinic?

- *[Probe based on responses to first set of questions]* global assessment, episodic and preventative care, health promotion, chronic disease management, pharmaceutical management, paediatric and women’s health, case management, care coordination, collaboration, and practice organization.

1. Who are other members of the primary care team? (e.g., number of other providers and roles)
2. How is your current position within the primary care setting funded?
   Does the physician pay your salary directly?
   Is your salary dependent on the physician’s salary or vice versa?
   Are you employed at the clinic full-time, part-time, or casually?

- *[Probe based on responses to first set of questions]* (Enhanced) Fee for Service, Capitation, globally-funded

1. How much of your time in the clinic is dedicated to patient care activities compared to non-patient care activities (e.g., administrative tasks, making appointments, cleaning, restocking)?
2. How did you and the physician/team arrive at an agreement about your role in the clinic?
3. How do you feel about your scope of practice in this role? How do you feel about the use of your knowledge/skills in this role?
4. What resources are available to you to help you work within this clinic?

- *[Probe based on responses to first set of questions]* meeting space, access to EMR, private office space

1. Do you feel that you are maximizing your potential contributions to patient care in this clinic? What do you find prevents/hinders you from optimizing your contribution to patient care within this clinic?
   If your role is already optimized, what are some facilitators?

- *[Probe based on responses to first set of questions]* *Barriers:* lack of time, cost of continuing education, inability to order diagnostic tests, lack of space, equipment or supplies, inability to prescribe/revise medications, lack of support from other providers, underutilized clinical skills, physicians attitudes towards role, patients perception of role, lack of educational opportunities, lack of clear job description; *Facilitators*: collaboration with physicians/other healthcare providers; access to education/training, positive patient feedback, supportive employment conditions, availability of space, equipment or supplies, ability to practice autonomously, awareness and access to programmes for clients, collaboration with community services

1. How does your health care team coordinate patient care? What supports are available to you to help carry out your roles? What are some barriers?

*[Probe based on responses to first set of questions]* improve access, continuity of care, patient satisfaction

1. Could you describe any efforts put into facilitating teamwork within your health care team? What are some barriers to optimizing teamwork?

*[Probe based on responses to first set of questions]* dedicated time for team meetings, team emails

1. Do you have any additional information that you would like to share?
